# Early *Clostridium difficile* Infection during Allogeneic Hematopoietic Stem Cell Transplantation

**DOI:** 10.1371/journal.pone.0090158

**Published:** 2014-03-24

**Authors:** Melissa A. Kinnebrew, Yeon Joo Lee, Robert R. Jenq, Lauren Lipuma, Eric R. Littmann, Asia Gobourne, Daniel No, Marcel van den Brink, Eric G. Pamer, Ying Taur

**Affiliations:** 1 Infectious Diseases Service, Memorial Sloan Kettering Cancer Center, New York, New York, United States of America; 2 Adult Bone Marrow Transplant Service, Memorial Sloan Kettering Cancer Center, New York, New York, United States of America; 3 Lucille Castori Center for Microbes, Inflammation, and Cancer, Memorial Sloan Kettering Cancer Center, New York, New York, United States of America; 4 Immunology Program, Sloan-Kettering Institute, New York, New York, United States of America; 5 Weill Cornell Medical College, New York, New York, United States of America; Beth Israel Deaconess Medical Center, Harvard Medical School, United States of America

## Abstract

*Clostridium difficile* infection (CDI) is frequently diagnosed in recipients of allogeneic hematopoietic stem cell transplantation (allo-HSCT). We characterized early-transplant CDI and its associations, and analyzed serially-collected feces to determine intestinal carriage of toxigenic *C. difficile*. Fecal specimens were collected longitudinally from 94 patients during allo-HSCT hospitalization, from the start of pre-transplant conditioning until up to 35 days after stem cell infusion. Presence of *C. difficile* 16S rRNA and *tcdB* genes was determined. Clinical variables and specimen data were analyzed for association with development of CDI. Historical data from an additional 1144 allo-HSCT patients was also used. Fecal specimens from 37 patients (39%) were found to harbor *C. difficile*. Early-transplant CDI was diagnosed in 16 of 94 (17%) patients undergoing allo-HSCT; cases were generally mild and resembled non-CDI diarrhea associated with transplant conditioning. CDI was associated with preceding colonization with tcdB-positive *C. difficile* and conditioning regimen intensity. We found no associations between early-transplant CDI and graft-versus-host disease or CDI later in transplant. CDI occurs with high frequency during the early phase of allo-HSCT, where recipients are pre-colonized with toxigenic C. difficile. During this time, CDI incidence peaks during pre-transplant conditioning, and is correlated to intensity of the treatment. In this unique setting, high rates of CDI may be explained by prior colonization and chemotherapy; however, cases were generally mild and resembled non-infectious diarrhea due to conditioning, raising concerns of misdiagnosis. Further study of this unique population with more discriminating CDI diagnostic tests are warranted.

## Introduction

*Clostridium difficile* infection (CDI) is a frequent cause of diarrhea in hospitalized patients, with disturbances of the intestinal microbiota following antibiotic administration as one of the major risk factors. Patients undergoing allogeneic hematopoietic stem cell transplantation (allo-HSCT) receive chemotherapy, radiation, and antibiotics, which represent significant risk factors for CDI. Previous studies demonstrated that approximately 15–30% of allo-HSCT recipients develop CDI during transplantation [1]–[5], greatly exceeding rates in most other patient populations.

Allo-HSCT patients have often been previously hospitalized, and each hospitalization increases exposure to *C. difficile*, providing a potential explanation for the high incidence of CDI. Although *C. difficile* can be acquired during hospitalization, prospective molecular typing of *C. difficile* isolates from hospitalized patients suggests that transmission may account for a minority of CDI cases, and that many patients who enter the hospital are colonized with *C. difficile* [6].

Previous studies have correlated CDI in allo-HSCT recipients with the development of graft-versus-host disease (GVHD). However, the rates of *C. difficile* colonization and the risk of CDI in colonized patients remain undefined in this population. Therefore, we examined the colonization status of patients over the course of early allo-HSCT, using a previously described cohort in which fecal specimens were collected throughout their transplant hospitalization. We also examined 13 years of observational data of allo-HSCT recipients cared for at our institution to supplement findings from our biospecimen cohort.

## Methods

### Biospecimen Protocol Group

Fecal specimens were collected from adult patients undergoing allo- HSCT at Memorial Sloan-Kettering Cancer Center. We developed a biospecimen collection protocol in which consenting patients underwent once weekly serial specimen collection during their transplant hospitalization, from up to 15 days pre-transplantation until up to 35 days post-transplantation. For each patient, specimen collection and study observation occurred within this 50-day window and while patients were still hospitalized for transplantation. For each subject we required that a minimum of one pre- and two post-transplant fecal specimens be collected for inclusion. Collection took place for patients with dates of transplantation from 4 September 2009 to 4 August 2011. This cohort of patients has been described in a previous report [7].

### Analysis of Fecal Specimens: *tcdB*

Fecal specimens collected from the biospecimen group were frozen and stored at −80°C upon collection until processed. DNA was purified from the stool specimens using a phenol-chloroform extraction process as previously described [8], [9]. DNA was purified further using QIAamp mini spin columns (Qiagen). Extracted DNA was analyzed by real-time PCR for the presence of *C. difficile* toxin B gene (*tcdB*). For the PCR reaction, 50 ng of extracted DNA was used as starting material along with 12.5 µL of Dynamo SYBR Green Master Mix (Thermo) and 400 nM of toxin B-specific primer sequences (Forward: GAAAGTCCAAGTTTACGCTCAAT, Reverse: GCTGCACCTAAACTTACACCA) [10]–[12].

Each specimen was run in duplicate. Real-time PCR was performed by the Step One Plus Real-Time PCR (Applied Biosystems). The PCR parameters were as follows: 94°C for 3 min and 30 cycles of 94°C for 30 sec, 52°C for 30 sec, and 72°C for 1 min. Amplification of bacterial 16S rRNA gene using universal primers was performed in parallel to ensure the specimen was not contaminated with PCR inhibitors (Forward: GCCAGCAGCCGCGGTAA and Reverse: AGGGTATCTAATCCT) [13],[14]. Melting curves of each reaction were examined and compared to positive controls to identify specific amplification.

For quantitation of *C. difficile* in the stool, primers specific for the *C. difficile* 16S rRNA gene were used in the same protocol described above (Forward: TTGAGCGATTTACTTCGGTAAAGA, Reverse: CCATCCTGTACTGGCTCACCT) [15]. Standard curves were prepared with known concentrations of a plasmid containing 1 copy of the *C. difficile* 16S rRNA gene.

### Observational Group

To complement the results from data in the biospecimen group, we gathered a larger dataset containing historical clinical data from medical records of patients undergoing allo-HSCT at our institution from 1 January 1999 to 29 March 2012, spanning approximately 13 years. To avoid analysis of duplicate data, patients included in the biospecimen group were excluded from the observational data group.

### Clinical Data

For both the biospecimen group and the observational group, clinical data were obtained for each patient through abstraction from electronic medical records. Data obtained included age, sex, underlying disease, conditioning regimen intensity [16], stem cell donor source, whether stem cell were ex-vivo T-cell depleted [17], antibiotics used during hospitalization (with start and stop dates), development of gastrointestinal graft-versus-host disease (GVHD) up to 100 days post-transplantation [18].

The diagnosis of CDI was based on a compatible clinical history with positive laboratory test, which was performed at the discretion of the transplant healthcare provider. Our microbiology laboratory only tested unformed stool for evidence of CDI. The severity of CDI cases was assessed using a three-tiered scoring system, described previously: low severity, medium severity, and severe disease [19].

The method of detection for toxin-producing *C. difficile* employed by our institution varied over the course of the study period. Prior to 29 August, 2008, a cell culture-based cytotoxicity assay (toxin and antitoxin reagents from TechLab, Wi-38 cell lines from Diagnostic Hybrids and Viromed Labs) was used. From 29 August, 2008 to 10 September, 2010, our hospital employed a two-step procedure involving detection of the GDH antigen (C. Diff Chek-60, Wampole Laboratories), followed by confirmatory cytotoxicity assay in GDH-positive specimens. On 10 September 2010, clinical CDI testing was switched to a PCR assay that detects *C. difficile* Toxin B (Xpert *C. difficile* assay, Cepheid GeneXpert), and continues to be used currently.

At our institution, routine antibiotic prophylaxis was given to patients undergoing allo-HSCT. Practice patterns varied slightly over the course of the study period, but were more formalized starting June 11, 2006. In general, intravenous vancomycin and ciprofloxacin were given to patients undergoing allo-HSCT with myeloablative or reduced intensity conditioning, from 2 days pre- to 7 days post-transplantation. Ciprofloxacin treatment could be longer, or for a non-myeloablative transplant, depending on anticipated time to engraftment. Our institution does not administer metronidazole for prevention of infection or GVHD.

### Ethics Statement

Specimen collection and analysis of the biospecimen group was approved by the Memorial Sloan-Kettering Cancer Center Institutional Review Board (IRB# 09-067). All biosepcimen group subjects provided written consent for specimen collection and analysis. For analysis of data from subjects from the observational group, we obtained an existing-data waiver from the Memorial Sloan-Kettering Cancer Center Institutional Review Board.

### Analytic Methods

Subjects within the biospecimen subset group were analyzed separately from the remaining observational cohort. Predictors of early transplant CDI were assessed using Cox proportional hazards regression, where predictors included clinical variables listed above, as well as preceding detection of *tcdB* for the biospecimen subset group. As secondary analyses, we also assessed the predictivity of early CDI onto the endpoints of late CDI and the development of gastrointestinal GVHD. We also assessed the risk factors for the presence of *tcdB* colonization in the first collected specimen, as an additional analysis.

Firth's penalized likelihood method was applied to all survival regression calculations, in order to avoid divergent parameter estimates due to monotone likelihood [20]. Since presence of *tcdB* and antibiotic administration were variables that changed over time, these predictors were coded and analyzed as time-dependent variables. In each of these analyses, predictors were analyzed separately in a univariate fashion; predictors with a univariate P-value less than or equal to 0.20 were analyzed in a multivariate model, to account for confounding influences. Survival plots for CDI were constructed using the Kaplan-Meier method. All analyses were performed using R version 3.01 (R Development Core Team, Vienna, Austria).

## Results

Fecal specimens were collected from 105 consented patients; 11 were excluded due to insufficient sample collection, leaving a total of 94 patients for study in the biospecimen group. From these subjects, a total of 436 fecal specimens were collected (3 to 8 per patient). Clinical characteristics of these patients are described in Table 1. During the observation period, 16 (17%) subjects were diagnosed with CDI. Among the remaining 78 subjects not diagnosed with CDI, an additional 21 patients had detectable *tcdB* by PCR. The approximate threshold of detection was 200 colony-forming units per gram of stool. We confirmed the presence of *C. difficile* in these specimens by quantitative PCR specific for *C. difficile* 16S rRNA genes. In patients diagnosed with CDI, a greater proportion of patients received myeloablative conditioning compared with those not diagnosed with CDI. Most patients diagnosed with CDI received treatment with metronidazole. Based on CDI severity scoring [19], cases were considered mild or moderate (13 low severity, 3 medium severity), with no cases of severe CDI.

**Table 1:** Characteristics of Patients, Biospecimen Group (N = 94)^a^.

Parameter	Diagnosed with CDI	Not Diagnosed with CDI		Total (Combined)
		*tcdB*+	*tcdB*−	
**Age (years)** g	50.5 (19–69)	57 (28–65)	54 (23–69)	53.5 (19–69)
**Sex (female)**	4 (25.0%)	10 (47.6%)	27 (47.4%)	41 (43.6%)
**Underlying Disease**				
**Leukemia**	11 (68.8%)	9 (42.9%)	24 (42.1%)	44 (46.8%)
**Lymphoma**	2 (12.5%)	6 (28.6%)	18 (31.6%)	26 (27.7%)
**Multiple Myeloma**	1 (6.2%)	3 (14.3%)	4 (7.0%)	8 (8.5%)
**Myelodysplastic Syndrome**	2 (12.5%)	1 (4.8%)	9 (15.8%)	12 (12.8%)
**Other**	0 (0.0%)	2 (9.5%)	2 (3.5%)	4 (4.3%)
**Prior antibiotics (14 d)** f	8 (50.0%)	7 (33.3%)	27 (47.4%)	42 (44.7%)
**Conditioning Regimen Intensity**				
**Non-myeloablative**	1 (6.2%)	5 (23.8%)	10 (17.5%)	16 (17.0%)
**Reduced Intensity**	2 (12.5%)	5 (23.8%)	14 (24.6%)	21 (22.3%)
**Myeloablative**	13 (81.2%)	11 (52.4%)	33 (57.9%)	57 (60.6%)
**T-cell depleted graft**	10 (62.5%)	10 (47.6%)	22 (38.6%)	42 (44.7%)
**Stem cell source (cord vs. other)**	3 (18.8%)	5 (23.8%)	15 (26.3%)	23 (24.5%)
**Time to engraftment (≥14 d)** b	3 (18.8%)	4 (19.0%)	17 (29.8%)	24 (25.5%)
**Antibiotics** c **^,^** d				
**Vancomycin (IV)**	15 (93.8%)	20 (95.2%)	50 (87.7%)	85 (90.4%)
**Fluoroquinolone**	3 (18.8%)	5 (23.8%)	25 (43.9%)	33 (35.1%)
**Metronidazole**	14 (87.5%)	3 (14.3%)	13 (22.8%)	30 (31.9%)
**Beta-lactams** e	13 (81.2%)	17 (81.0%)	52 (91.2%)	82 (87.2%)
**Number of Specimens Collected** g				
**Pre-transplant**	2 (1–2)	2 (1–2)	1 (1–3)	2 (1–3)
**Post-transplant**	3 (2–6)	3 (2–7)	3 (2–5)	3 (2–7)
**Total**	**16 (100.0%)**	**21 (100.0%)**	**57 (100.0%)**	**94 (100.0%)**

a Characteristics of patients in the observational group (N = 1144) can be found in Table S1.

b Engraftment was defined as an absolute neutrophil count greater than 500 cells/µL for three consecutive days.

c Assessed during inpatient allo-HSCT hospitalization, from beginning of pre-transplant up to 35 days post-transplant.

d Antibiotics are not mutually exclusive categories and thus does not sum to 100%.

e Beta-lactams include cephalosporins, beta-lactam/beta-lactamase combinations, and carbapenems.

f Prior antibiotics refer to antibiotics given prior to allo-HSCT and prior to observation time, within 14 days.

g Reported as median, with range in parentheses.

**Figure 1:**
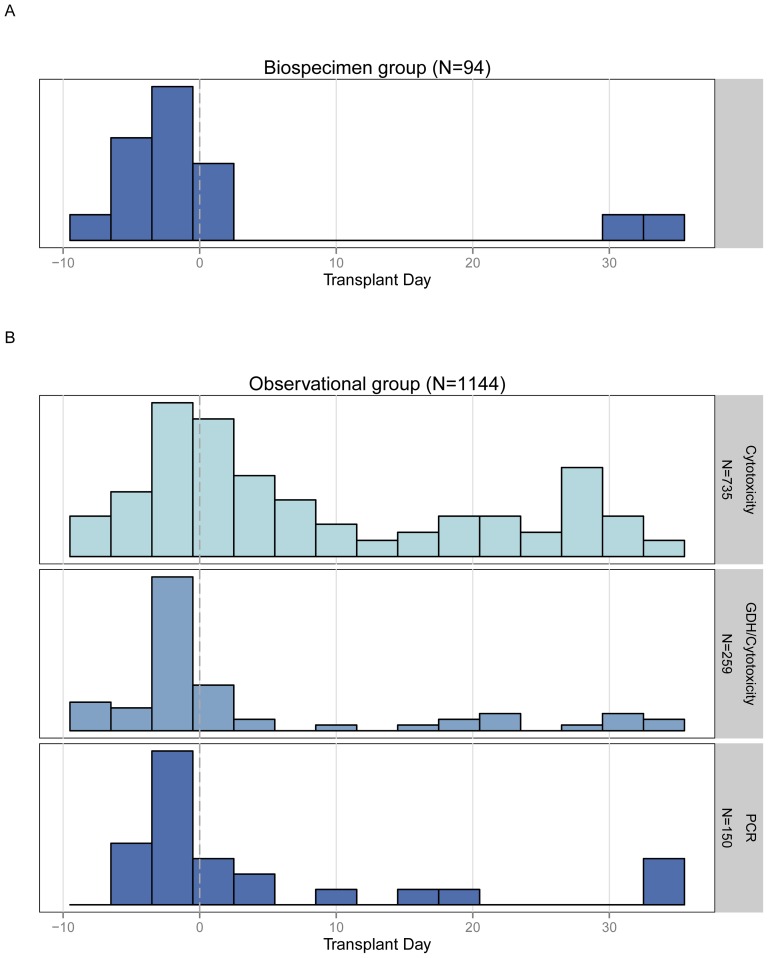
Histogram of early CDI cases by transplant day. Cases peaked approximately on or slightly prior to stem cell infusion. This pattern was evident in both subject groups and regardless of CDI testing method. *A*, Biospecimen group (N = 94). *B*, Observational group (N = 1144).

Within the observational data group, a total of 1144 subjects were included. CDI was diagnosed in 138 patients (12.1%), and showed similar clinical characteristics as the biospecimen group (Table S1). In both the biospecimen and observational groups, most cases of CDI occurred in the immediate peri-transplant period (i.e. within a few days of stem cell infusion), peaking just prior to stem cell infusion (Figure 1). This pattern of distribution over relative day of transplant was observed regardless of CDI testing method, though the overall CDI rate in this population increased over time (Figure S1).

**Figure 2:**
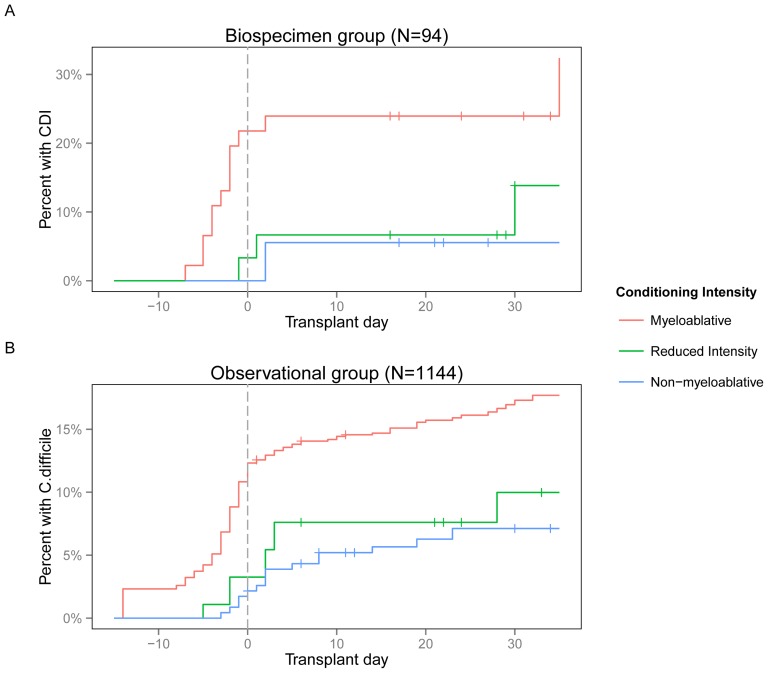
Kaplan-Meier plot of CDI during allo-HSCT. Patients receiving greater intensity conditioning regimens were more likely to develop CDI. *A*, Biospecimen group (N = 94). *B*, Observational group (N = 1144).

Analysis of risk factors for CDI are listed in Table 2. Myeloablative conditioning increased the risk of CDI three-fold in comparison to regimens of lower intensity (Figure 2). These findings are consistent with recent studies describing CDI in patients following allo-HSCT [2], [5]. In addition to conditioning, the observational group showed that an underlying diagnosis of leukemia and ex-vivo T-cell depletion were also associated with CDI, and beta-lactam administration was protective against subsequent CDI. Analysis of the biospecimen group demonstrated that preceding colonization with *tcdB-*positive *C. difficile* was highly predictive of subsequent diagnosis of CDI. On multivariate analysis, conditioning regimen, prior colonization in the biospecimen group, and beta-lactam administration in the observational group remained as independent predictors (Table S2 and S3). A follow-up analysis of predictors of toxigenic *C. difficile* colonization within the first collected specimen showed no significant associations (Table S4).

**Table 2:** Univariate predictors of CDI in the biospecimen group and observational group, by Cox proportional hazards regression.

Predictor	Biospecimen group (N = 94)^a^		Observational Group (N = 1144)^b^	
	Haz ratio (95% CI)	P	Haz ratio (95% CI)	P
**Age (years)**	0.98 (0.94–1.02)	0.311	1.01 (0.99–1.02)	0.279
**Sex (female)**	0.41 (0.12–1.14)	0.089	1.02 (0.72–1.42)	0.928
**Underlying Disease (leukemia vs. other)**	2.44 (0.92–7.35)	0.075	1.42 (1.01–2.02)	0.044
**Conditioning Regimen (myeloablative vs. other)**	3.18 (1.14–10.63)	0.026	1.99 (1.30–3.17)	0.001
**T-cell depleted graft**	1.96 (0.72–5.51)	0.185	1.59 (1.14–2.24)	0.007
**Stem cell source (cord vs. other)**	0.49 (0.11–1.62)	0.263	1.31 (0.74–2.16)	0.331
**Prior antibiotics (14 days)** f	1.31 (0.50–3.45)	0.580		
**Antibiotics** c				
**Vancomycin (IV)**	3.16 (0.85–11.91)	0.085	0.79 (0.53–1.18)	0.242
**Metronidazole**	0.43 (0.00–3.63)	0.518	0.73 (0.41–1.24)	0.252
**Fluoroquinolones** d	0.28 (0.03–1.44)	0.139	0.90 (0.60–1.33)	0.605
**Beta-lactam** e	1.28 (0.40–3.78)	0.666	0.67 (0.45–1.00)	0.048
***tcdB*** ** positivity** c	17.16 (6.40–51.97)	0.000		

a Multivariate analysis of the biospecimen group can be found in Table S2.

b Multivariate analysis of the observational group can be found in Table S3.

c Analyzed as a time-varying predictor.

d Fluoroquinolones consist of ciprofloxacin and levofloxacin.

e Beta-lactams include cephalosporins, beta-lactam/beta-lactamase combinations, and carbapenems.

f Prior antibiotics refer to antibiotics given prior to allo-HSCT and prior to observation time, within 14 days, and were not analyzed as time-varying predictors.

**Figure 3:**
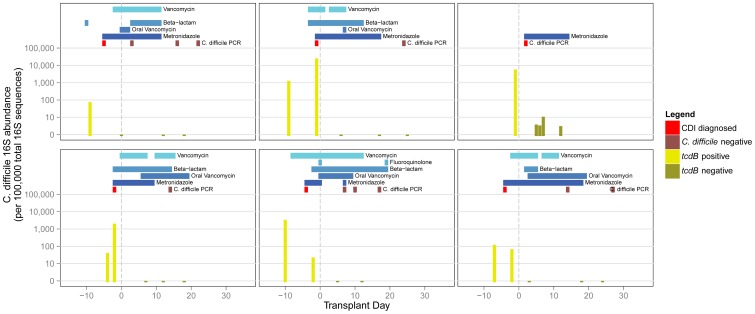
Intestinal composition of *C. difficile* in six selected cases of CDI. Observed abundance of C. difficile 16S is shown for each fecal specimen in the bargraph over the course of transplantation. The corresponding timing of CDI and antibiotic administration is shown at the top of each plot. Plots of all 94 patients in the biospecimen group can be found in Figure S1.

Selected patients diagnosed with CDI are shown in Figure 3 (plots of all patients are listed in Figure S2). *C. difficile* 16S was detectable in all *tcdB*-positive specimens. Conversely, there were some specimens in which *C. difficile* 16S was detectable, but were *tcdB*-negative. These specimens may represent non-toxigenic strains of *C. difficile* or closely related species. Patients diagnosed with CDI often had preceding colonization by *tcdB*-positive *C. difficile*. In almost all cases, *tcdB* became undetectable upon initiation of treatment with metronidazole.

**Figure 4:**
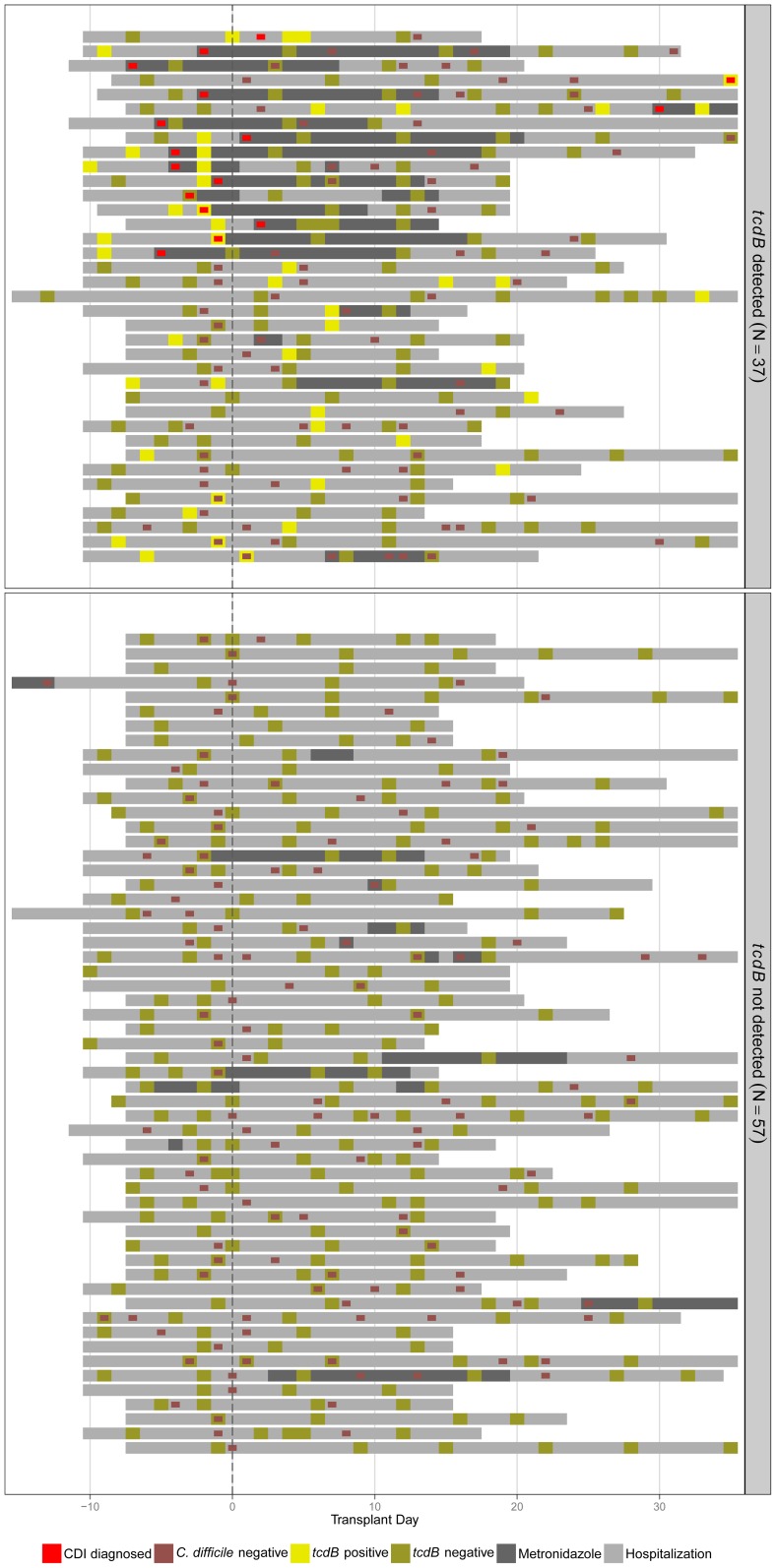
*C. difficile* colonization status in the biospecimen group subjects (N = 94) over the course of allo-HSCT. Each row represents one subject during hospitalization for allo-HSCT. Yellow squares represent results of *tcdB* testing of fecal specimens; red squares represent clinical testing for CDI. Dark shading shows metronidazole administration.

*C. difficile* colonization status over the course of transplant is shown for each patient in Figure 4. Colonization was detectable in 37 out of 94 patients (39%). In these patients, *tcdB* positivity was intermittent over the course of hospitalization, but was often detected near the start of hospitalization. Of these subjects, 16 (43%) were clinically diagnosed with CDI within the study period and treated. In two cases of CDI, prior fecal specimens were negative for *tcdB* colonization (3% of all previously non-colonized); three subjects did not have evaluable specimens prior to CDI diagnosis.

During times of treatment with metronidazole, *tcdB* was typically undetectable. Only two CDI cases were diagnosed beyond three days after stem cell infusion, while nine CDI cases occurred within 72 hours of stem cell infusion. 15 of 16 patients were diagnosed by PCR while one was diagnosed by cytotoxicity testing.

We analyzed early-transplant CDI with subsequent clinical endpoints within the biospecimen cohort; we found no detectable associations with gastrointestinal GVHD (HR 0.45, 95% CI 0.05–1.81) or CDI later in the course of transplantation (HR 2.44, 95% CI 0.71–7.16).

## Discussion

CDI continues to be a significant concern in recipients of allo-HSCT. In this study we observed a high rate of CDI during conditioning and the first month following transplantation, occurring in 17% of our biospecimen group and 12.1% of our observational group. Similar CDI rates have been described for allo-HSCT recipients at other centers [1]–[5]. We found CDI to be mild to moderate in severity and temporally associated with allo-HSCT conditioning. We and others have observed that a large proportion of cases occur during the early allo-HSCT period, prior to stem cell engraftment when patients are neutropenic [1], [2]. In this study we further characterized CDI during the first month following allo-HSCT by prospective fecal specimen analysis.

Clinically, we found that the diagnosis of early transplant CDI was common and patients appeared to respond rapidly to antibiotic therapy. Early allo-HSCT CDI correlated with high intensity chemotherapy regimens, but not with antibiotic administration. It is noteworthy that most cases of CDI occurred prior to hematopoietic stem cell infusion. This early in the course of transplantation, patients have not yet undergone hematopoietic stem cell infusion, and many have only received prophylactic antibiotics thus far. Though there can be exceptions, risk of bloodstream infection and the corresponding empiric treatment with broad-spectrum antibiotics generally come later, and peak several days after stem cell infusion. Therefore it could be that CDI in this setting arises largely as a result of chemotherapy and radiation that is given as part of the conditioning regimen, and less to antibiotic administration. Our observed association with conditioning regimen intensity would seem to support this. Several factors we examined, including stem cell characteristics and antibiotic administration, may have occurred largely after the peak of CDI. Though we performed a time-dependent analysis for some factors in order to avoid survival bias, this might explain why these factors were not significantly associated. We observed that T-cell depletion was a significant univariate risk factor in our observational cohort; this association is more likely related to associated pre-transplant confounders, rather than to T-cell depletion itself. Indeed, this became non-significant in the multivariate model. We repeated the analysis with observation time for CDI starting at the time of stem cell infusion, and did not find any additional significant predictors of CDI (results not shown).

Within our biospecimen cohort, we found that 39% of patients harbored toxigenic *Clostridium difficile* based on PCR detection of *tcdB*, revealing a high rate of colonization in these patients. Patients in this study who ultimately developed CDI were generally pre-colonized, whereas CDI in a previously non-colonized patient was rare. Though our study did not focus on pre-transplantation events, we did not detect any clear predictors of pre-colonization itself. A high colonization rate with toxigenic *C. difficile*, combined with disruption of intestinal microbiota and intestinal epithelial barriers by intense myeloablative conditioning may, at least in part, explain the high rates of CDI observed in this population.

Alternatively, however, it is possible that CDI is misdiagnosed during early stages of allo-HSCT. Most CDI diagnoses were made when diarrhea resulting from pre-transplant conditioning is common. In allo-HSCT patients diagnosed with CDI, diarrhea was usually mild and essentially indistinguishable from conditioning-related diarrhea. At our institution, diarrhea during transplantation is extremely common. Using this study's data as one estimate, fecal specimens were submitted for clinical testing in 95% (89/94) of patients in our biospecimen cohort and 84% (960/1144) of our observational cohort, suggesting a high rate of diarrhea. Other centers have also reported high rates of diarrhea [21]. False positivity, in the setting of a high colonization rate, combined with an inherent testing bias around the time of stem cell infusion, might explain the high frequency of CDI diagnoses during the early transplant period and could also explain the association of CDI with intense conditioning. Also consistent with this is the observation that CDI during early allo-HSCT was not predictive of subsequent CDI at later time points in the post-transplantation period.

If true, then it is possible that the CDI rate reported by our institution and other transplant centers is overestimated. The recent introduction of PCR assays to diagnose CDI may increase the risk for false positivity, since PCR does not distinguish between CDI and asymptomatic colonization [22]. Thus, *C. difficile* PCR assays may be particularly problematic in patient populations with high colonization rates and alternative causes of diarrhea. Improved methods for detection hold some promise to enhance the specificity of CDI diagnosis. For instance, use of a functional cytotoxicity assay [23] that detects and quantifies toxin (similar to the cytotoxicity assay) could demonstrate active toxin expression, presumably a better indicator of disease, rather than simply demonstrating the presence of the gene encoding the *C. difficile* toxin.

In this study, metronidazole treatment appeared to inhibit detectable toxigenic *C. difficile*. However, this may not reflect complete elimination, since our method of detection was not optimized to detect *C. difficile* spores. This form is resistant to antibiotics, and may very well be linked to the pathogenesis of recurrent CDI infections [24]. At our institution, early CDI was typically treated with metronidazole. Oral vancomycin and fidaxomycin are alternative agents which may be preferred agents for moderate-to-severe CDI or recurrences [25], [26], but our study suggests that CDI during early allo-HSCT is generally mild and does not predispose to CDI later in the course of transplant. Therefore in this particular clinical scenario, metronidazole may be sufficiently efficacious compared with other *C. difficile* agents.

However, unnecessary treatment of *C. difficile*-colonized patients is not inconsequential. Metronidazole is associated with subsequent intestinal domination and bloodstream infection by vancomycin-resistant *Enterococcus* (VRE), independent of conditioning intensity [7]. Other studies have also demonstrated that metronidazole and other antibiotics with anti-anaerobic activity are potent promoters of dense intestinal VRE colonization [27]–[29]. Furthermore, prior studies demonstrated that colonization with toxigenic or non-toxigenic *C. difficile* with CDI can be protective [30]–[32]. Fidaxomicin has a narrower spectrum of activity and may be less likely to promote VRE colonization [33]; it could be that this treatment might be preferred for early transplant CDI, given the consequences of a perturbed microbiota in this population [7], [34].

Several studies have correlated CDI with GVHD [2], [4], [5], [35], raising the possibility that prevention of CDI might reduce the risk of GVHD. However, we did not detect an association between CDI during the first month following allo-HSCT and subsequent GVHD. There are several possible explanations for this disparity. For example, in the subset of patients undergoing T-cell depleted allo-HSCT, ex-vivo T-cell depletion of hematopoietic stem cell grafts prior to infusion results in a markedly lower incidence of GVHD, which might reduce statistical power and impair our ability to detect an association. Alternatively, there were some notable differences in analytic methodology. We analyzed CDI as a time-dependent predictor for GVHD as an endpoint, in order to obtain an unbiased estimate using only CDI occurring prior to onset of GVHD. Of the prior studies, only two performed survival analysis [2], [5], and of those, only one utilized a time-dependent analysis, and in that study the predictor and endpoint were switched: preceding GVHD was examined as a risk factor for subsequent CDI [2]. Finally, yet another possibility is that, similar to the association with high intensity chemotherapy, the observed association between CDI and GVHD may be explained by an inherent bias in testing.

In conclusion, we find that CDI is frequently diagnosed during early allo-HSCT, particularly using PCR detection. Given the high frequency of diarrhea in patients receiving high-intensity allo-HSCT conditioning, the risk of false positivity is unknown but potentially significant. Thus, uncertainty as to the true CDI rate in allo-HSCT patients remains, and distinguishing CDI from diarrhea associated with pre-transplant conditioning or graft-versus-host disease continues to be a major clinical challenge. Given the high rate of colonization and intensive treatments with antibiotics, chemotherapy, and immunosuppressants, CDI should continue to remain a concern in recipients of allo-HSCT, but further study and application of better diagnostic strategies will be required to restrict CDI treatment to only those patients with *C. difficile* toxin-mediated colitis.

## Supporting Information

Figure S1 Incidence of Clostridium difficile infection within observation period, in entire study cohort (biospecimen+observational group, N = 1238). Expressed as percentage of transplant recipients.
(TIFF)

Figure S2 Intestinal composition of C. difficile in the biospecimen group (N = 94). Fecal specimens are barplotted over transplant day. The timing of C. difficile testing and antibiotic administration is shown at the top of each plot.
(TIF)

Table S1 Characteristics of Patients, Observational Group (N = 1144).
(DOC)

Table S2 Multivariate predictors of CDI in biospecimen group (N = 94).
(DOC)

Table S3 Multivariate predictors of CDI in observational group (N = 1144).
(DOC)

Table S4 Predictors of toxigenic *Clostridium difficile* colonization, by fecal detection of tcdB (N = 94).
(DOC)
