# Supplementary material for: Early Clostridium difficile Infection during Allogeneic Hematopoietic Stem Cell Transplantation
Source: PLoS One. 2014 Mar 24;9(3):e90158. doi: 10.1371/journal.pone.0090158 (PMC3963842; doi:10.1371/journal.pone.0090158)
Supplement: Table S1 — Characteristics of Patients, Observational Group (N = 1144). (DOC) [file pone.0090158.s003.doc]

Table S1: Characteristics of Patients, Observational Group (N=1144)

| **Parameter** | **Diagnosed with CDI** | **Not Diagnosed with CDI** | **Total** |
| --- | --- | --- | --- |
| **Age (years)^e^** | 49 (18-69) | 47 (18-73) | 47 (18-73) |
| **Sex (female)** | 58 (42.0%) | 418 (41.6%) | 476 (41.6%) |
| **Underlying Disease** |  |  |  |
| **Leukemia** | 87 (63.0%) | 534 (53.1%) | 621 (54.3%) |
| **Lymphoma** | 21 (15.2%) | 242 (24.1%) | 263 (23.0%) |
| **Multiple Myeloma** | 4 (2.9%) | 41 (4.1%) | 45 (3.9%) |
| **Myelodysplastic Syndrome** | 23 (16.7%) | 132 (13.1%) | 155 (13.5%) |
| **Other** | 3 (2.2%) | 57 (5.7%) | 60 (5.2%) |
| **Conditioning Regimen** |  |  |  |
| **Non-myeloablative** | 15 (10.9%) | 219 (22.5%) | 234 (21.0%) |
| **Reduced Intensity** | 8 (5.8%) | 84 (8.6%) | 92 (8.3%) |
| **Myeloablative** | 115 (83.3%) | 672 (68.9%) | 787 (70.7%) |
| **T-cell depleted graft** | 75 (54.3%) | 437 (43.4%) | 512 (44.8%) |
| **Stem cell source (cord vs. other)** | 15 (10.9%) | 83 (8.3%) | 98 (8.6%) |
| **Time to engraftment (≥14d)Error: Reference source not found** | 35 (25.4%) | 284 (28.2%) | 319 (27.9%) |
| **AntibioticsError: Reference source not found** |  |  |  |
| **Vancomycin (IV)** | 131 (94.9%) | 931 (92.5%) | 1062 (92.8%) |
| **Fluoroquinolone** | 97 (70.3%) | 712 (70.8%) | 809 (70.7%) |
| **Metronidazole** | 129 (93.5%) | 417 (41.5%) | 546 (47.7%) |
| **Beta-lactamError: Reference source not found** | 131 (94.9%) | 935 (92.9%) | 1066 (93.2%) |
| **Total** | 138 (100.0%) | 1006 (100.0%) | 1144 (100.0%) |

^a^Engraftment was defined as an absolute neutrophil count greater than 500 cells/µL for three consecutive days.

^b^Antibiotics are not mutually exclusive categories and thus does not sum to 100%

^c^Beta-lactams include cephalosporins, beta-lactam/beta-lactamase combinations, and carbapenems.

^d^Assessed during inpatient allo-HSCT hospitalization, from beginning of pre-transplant up to 35 days post-transplant.

^e^Reported as median, with range in parentheses.
