# Supplementary material for: Early Clostridium difficile Infection during Allogeneic Hematopoietic Stem Cell Transplantation
Source: PLoS One. 2014 Mar 24;9(3):e90158. doi: 10.1371/journal.pone.0090158 (PMC3963842; doi:10.1371/journal.pone.0090158)
Supplement: Table S2 — Multivariate predictors of CDI in biospecimen group (N = 94). (DOC) [file pone.0090158.s004.doc]

Table S2: Multivariate predictors of CDI in biospecimen group (N=94)

| **Predictor** | **Univariate** | |  | **Multivariate** | |
| --- | --- | --- | --- | --- | --- |
|  | **Haz ratio** | **P-value** |  | **Haz ratio** | **P-value** |
| **Age (years)** | 0.98 (0.94 - 1.02) | 0.311 |  |  |  |
| **Sex (female)** | 0.41 (0.12 - 1.14) | 0.089 |  | 0.18 (0.05 - 0.56) | 0.003 |
| **Underlying Disease (leukemia vs. other)** | 2.44 (0.92 - 7.35) | 0.075 |  | 1.06 (0.36 - 3.49) | 0.912 |
| **Conditioning Regimen (myeloablative vs. other)** | 3.18 (1.14 - 10.63) | 0.026 |  | 9.51 (1.87 - 53.06) | 0.007 |
| **T-cell depleted graft** | 1.96 (0.72 - 5.51) | 0.185 |  | 0.44 (0.12 - 1.79) | 0.235 |
| **Stem cell source (cord vs. other)** | 0.49 (0.11 - 1.62) | 0.263 |  |  |  |
| **Prior antibiotics (14 days)^d^** | 1.31 (0.50 - 3.45) | 0.580 |  |  |  |
| **Antibioticsa** |  |  |  |  |  |
| **Vancomycin (IV)** | 3.16 (0.85 - 11.91) | 0.085 |  | 1.03 (0.26 - 4.41) | 0.969 |
| **Metronidazole** | 0.43 (0.00 - 3.63) | 0.518 |  |  |  |
| **Fluoroquinoloneb** | 0.28 (0.03 - 1.44) | 0.139 |  | 0.42 (0.04 - 2.47) | 0.357 |
| **Beta-lactamc** | 1.28 (0.40 - 3.78) | 0.666 |  |  |  |
| ***tcdB* positivitya** | 17.16 (6.40 - 51.97) | 0.000 |  | 19.85 (6.34 - 76.64) | 0.000 |

^a^Analyzed as a time-varying predictor

^b^Fluoroquinolones consist of ciprofloxacin and levofloxacin

^c^Beta-lactams include cephalosporins, beta-lactam/beta-lactamase combinations, and carbapenems.

^d^Prior antibiotics refer to antibiotics given prior to allo-HSCT and prior to observation time, within 14 days.
