# Supplementary material for: Early Clostridium difficile Infection during Allogeneic Hematopoietic Stem Cell Transplantation
Source: PLoS One. 2014 Mar 24;9(3):e90158. doi: 10.1371/journal.pone.0090158 (PMC3963842; doi:10.1371/journal.pone.0090158)
Supplement: Table S3 — Multivariate predictors of CDI in observational group (N = 1144). (DOC) [file pone.0090158.s005.doc]

Table S3. Multivariate predictors of CDI in observational group (N=1144)

| **Predictor** | **Univariate** | |  | **Multivariate** | |
| --- | --- | --- | --- | --- | --- |
|  | **Haz ratio** | **P-value** |  | **Haz ratio** | **P-value** |
| **Age (years)** | 1.01 (0.99 - 1.02) | 0.279 |  |  |  |
| **Sex (female)** | 1.02 (0.72 - 1.42) | 0.928 |  |  |  |
| **Underlying Disease (leukemia vs. other)** | 1.42 (1.01 - 2.02) | 0.044 |  | 1.18 (0.82 - 1.72) | 0.366 |
| **Conditioning Regimen (myeloablative vs. other)** | 1.99 (1.30 - 3.17) | 0.001 |  | 1.72 (1.03 - 2.94) | 0.038 |
| **T-cell depleted graft** | 1.59 (1.14 - 2.24) | 0.007 |  | 1.28 (0.89 - 1.87) | 0.184 |
| **Stem cell source (cord vs. other)** | 1.31 (0.74 - 2.16) | 0.331 |  |  |  |
| **Antibioticsa** |  |  |  |  |  |
| **Vancomycin (IV)** | 0.79 (0.53 - 1.18) | 0.242 |  |  |  |
| **Metronidazole** | 0.73 (0.41 - 1.24) | 0.252 |  |  |  |
| **Fluoroquinoloneb** | 0.90 (0.60 - 1.33) | 0.605 |  |  |  |
| **Beta-lactamc** | 0.67 (0.45 - 1.00) | 0.048 |  | 0.62 (0.41 - 0.92) | 0.018 |

^a^Analyzed as a time-varying predictor

^b^Fluoroquinolones consist of ciprofloxacin and levofloxacin

^c^Beta-lactams include cephalosporins, beta-lactam/beta-lactamase combinations, and carbapenems.
