# Supplementary material for: Early Clostridium difficile Infection during Allogeneic Hematopoietic Stem Cell Transplantation
Source: PLoS One. 2014 Mar 24;9(3):e90158. doi: 10.1371/journal.pone.0090158 (PMC3963842; doi:10.1371/journal.pone.0090158)
Supplement: Table S4 — Predictors of toxigenic Clostridium difficile colonization, by fecal detection of tcdB (N = 94). (DOC) [file pone.0090158.s006.doc]

Table S4. Predictors of toxigenic *Clostridium difficile* colonization, by fecal detection of tcdB (N=94)

| **Predictor** | **Univariate** | |
| --- | --- | --- |
|  | **Haz ratio** | **P-value** |
| **Age (years)** | 1.02 (0.99 - 1.05) | 0.251 |
| **Sex (female)** | 0.77 (0.37 - 1.56) | 0.476 |
| **Underlying Disease (leukemia vs. other)** | 1.14 (0.56 - 2.31) | 0.709 |
| **Conditioning Regimen (myeloablative vs. other)** | 0.87 (0.42 - 1.80) | 0.713 |
| **Prior antibiotics (14 days)** | 0.69 (0.32 - 1.40) | 0.309 |
